# Unveiling the structure-emulsifying function relationship of truncated recombinant forms of the SA01-OmpA protein opens up a new vista in bioemulsifiers

**DOI:** 10.1128/spectrum.03465-23

**Published:** 2024-01-11

**Authors:** Naeema Mohseni Sani, Mahbubeh Talaee, Ali Akbari, Faranak Ashoori, Javad Zamani, Ali A. Kermani, Hossein Shahbani Zahiri, John Presley, Hojatollah Vali, Kambiz Akbari Noghabi

**Affiliations:** 1 Department of Energy and Environmental Biotechnology, National Institute of Genetic Engineering and Biotechnology (NIGEB), Tehran, Iran; 2 Department of Structural Biology, St. Jude Children’s Research Hospital, Memphis, Tennessee, USA; 3 Department of Anatomy & Cell Biology, McGill University, Montreal, Québec, Canada; Ocean University of China, Qingdao, China

**Keywords:** SA01-OmpA, truncated forms, NT-OmpA, CT-OmpA, emulsifying activity, cytotoxicity studies, bioemulsifier

## Abstract

The emulsifying ability of SA01-OmpA (outer membrane protein A from *Acinetobacter* sp. SA01) was found to be constrained by challenges like low production efficiency and high costs associated with protein recovery from *E. coli* inclusion bodies, as described in our previous study. The present study sought to benefit from the advantages of the targeted truncating of SA01-OmpA protein, taking into account the reduced propensity of protein expression as inclusion bodies and cytotoxicity. Here, the structure and activity relationship of two truncated recombinant forms of SA01-OmpA protein was unraveled through a hybrid approach based on experimental data and computational methodologies, representing an innovative bioemulsifier with advantageous emulsifying activity. The recombinant truncated SA01-OmpA variants were cloned and heterologously expressed in *E. coli* host cells and subsequently purified. The results showed increased emulsifying activity of N-terminally truncated SA01-OmpA (NT-OmpA) compared to full-length SA01-OmpA. Molecular dynamics (MD) simulations analysis demonstrated a direct correlation between the C-terminally truncated SA01-OmpA (CT-OmpA) and its expression as inclusion bodies. Analysis of the structure-activity relationship of truncated variants of SA01-OmpA revealed that, compared to the full-length protein, deletion of the β-barrel portion from the N-terminal of SA01-OmpA increased the emulsifying activity of NT-OmpA while lowering its expression as inclusion bodies. Contrary to the full-length protein, the N-terminally truncated SA01-OmpA was not as cytotoxic, according to the MTT assay, FCM analysis, and AO/EB staining. The findings of this extensive study advance our knowledge of SA01-OmpA at the molecular level as well as the design and development of efficient bioemulsifiers.

**IMPORTANCE:**

Previous research (Shahryari et al. 2021, mSystems 6: e01175-20) introduced and characterized the SA01-OmpA protein as a multifaceted protein with a variety of functions, including maintaining cellular homeostasis under oxidative stress conditions, biofilm formation, outer membrane vesicles (OMV) biogenesis, and beneficial emulsifying capacity. By truncating the SA01-OmpA protein, the current study presents a unique method for developing protein-type bioemulsifiers. The findings indicate that the N-terminally truncated SA01-OmpA (NT-OmpA) has the potential to fully replace full-length SA01-OmpA as a novel bioemulsifier with significant emulsifying activity. This study opens up a new frontier in bioemulsifiers, shedding light on a possible relationship between the structure and activity of SA01-OmpA truncated forms.

## INTRODUCTION

Outer membrane proteins (OMPs) serve a variety of biological functions, including intracellular communication, a transporter for selective passage of molecules, metabolite exchange, environmental sensing, and defense against host immunity (1). They are predominantly found in the outer membrane (OM) of mitochondria, chloroplasts, and Gram-negative bacteria (GNB) (2). OMPs typically have a β-barrel shape and can be monomeric or trimeric (3), and this structural characteristic aids bacterial resistance to adverse environmental conditions (4). OmpA, OmpW, and OmpX are members of the OMPs subgroup (1). The OmpA family is a subgroup of OMPs with surface-exposed and heat-modifiable characteristics. The majority of Gram-negative bacteria have a high copy number of OmpA, which is present in their outer membrane (5). The salt-induced electrostatic gating mechanism that regulates the ion pore of OmpA, which has both structural and ion-permeable porin activities, might aid bacterial survival under osmotic stress (6). From a structural standpoint, it is a multidomain protein with a transmembrane β-barrel at its N-terminus and a globular periplasmic domain at its C-terminus connected by a structureless loop (7). OmpA has been intensively studied in Gram-negative bacteria due to its importance in the adhesion of pathogens to host cells and its appropriate emulsifying ability (8). For example, in the hospital strain *Acinetobacter baumannii*, OmpA as a multifunctional virulence factor plays an important role in the biogenesis of outer membrane vesicles, biofilm formation, and resistance to different types of antimicrobial drugs (9). AlnA, a 349 amino acid protein produced by the environmental strain *Acinetobacter radioresistens* strain KA53, has obvious sequence similarity with OmpA from *Escherichia coli* and OprF, the major porin from *Pseudomonas aeruginosa*, and is responsible for the emulsifying activity of the Alasan complex (10). Due to their practical significance, research on developing harmless and ecologically friendly bioactive compounds, particularly bioemulsifiers, has increased dramatically in recent years (11–14). Bioemulsifiers are a class of substances whose actions are intimately tied to their chemical composition (15). Furthermore, bioemulsifiers present a versatile and environmentally friendly substitute to synthetic emulsifiers, with applications in a variety of industrial sectors, including food, medicine, and cosmetics (16). The high molecular weight and hydrophobicity of proteins influence how well they interact with cell membranes and how cytotoxic they are (17, 18). Owing to their unique properties, such as the capacity to stabilize oil-in-water emulsions and compatibility with environmental processes, interest in the research and use of bioemulsifiers has increased widely. Designing nanoemulsions to serve as emulsion-based delivery methods in the food industry has attracted more attention recently, as their tiny size results in improved stability and increased bioavailability (19). *Acinetobacter* sp. SA01, with a high capacity for phenol degradation, was previously characterized, and the structural and functional properties of an outer membrane protein A (OmpA) from this native bacterial strain were thoroughly described (20). Further research showed that this protein could be released to the extracellular environment through outer membrane vesicles (OMVs) and play a role in biofilm formation, like its homolog in *Acinetobacter baumannii*. The high expression of SA01-OmpA in cells fed with ethanol and phenol showed that oxidative stress could be a significant factor in regulating SA01-OmpA gene expression, which indicates its role in cellular homeostasis. Recombinant SA01-OmpA protein expressed in *E. coli* was purified and showed favorable emulsifying activity (10). In the following, it became clear that many challenges including low yield, high purification costs, and high cytotoxicity must be overcome to produce and use the multifunctional SA01-OmpA outer membrane protein as a bioemulsifier.

Protein truncation, also referred to as premature protein termination brought on by a stop codon in a structural gene, is the elimination of a protein’s N- or C-terminal portion through proteolysis or structural gene manipulation (21). Protein truncating variants (PTVs) are genetic variants that alter transcription, leading to shorter proteins that may have implications on either protein loss or gain of function (22). Some proteins have been known to undergo truncation modifications, such as the glucose-dependent insulinotropic polypeptide (GIP), which could serve as a stronger antagonist for GIP receptors (23). According to a previous work by Grundmeier et al. (24), truncating fibronectin-binding proteins, involved in the pathogenesis of *Staphylococcus aureus*, results in loss of cell wall anchoring activity, hindering host cell attachment and invasion (24). Many pathogenic bacteria have developed complex coping mechanisms to deal with challenging environmental conditions. The development of small colony variations (SCVs), slow-growing subpopulations, is one tactic. In a prior study, *Salmonella enterica* with a truncated glutamine synthetase (*glnA*) gene exhibited a small colony variant phenotype, impaired host cell entry, and decreased the expression of flagellin and SPI-1-associated effector genes (25).

The main objective of this study was to gain a deeper grasp of the relationship between the structure and emulsifying abilities of two of SA01-OmpA’s truncated variants: the C-terminally truncated SA01-OmpA (CT-OmpA) (176 amino acids) and the N-terminally truncated SA01-OmpA (NT-OmpA) (166 amino acids). For that, the truncated recombinant proteins were cloned, expressed in *E. coli*, and purified to examine their emulsifying performance compared to the full-length protein. The full-length SA01-OmpA protein and its recombinant truncated variants were both subjected to molecular dynamics (MD) modeling and cytotoxicity assays.

## RESULTS

### Challenges in producing the bioemulsifier protein SA01-OmpA

**Fig 1:**
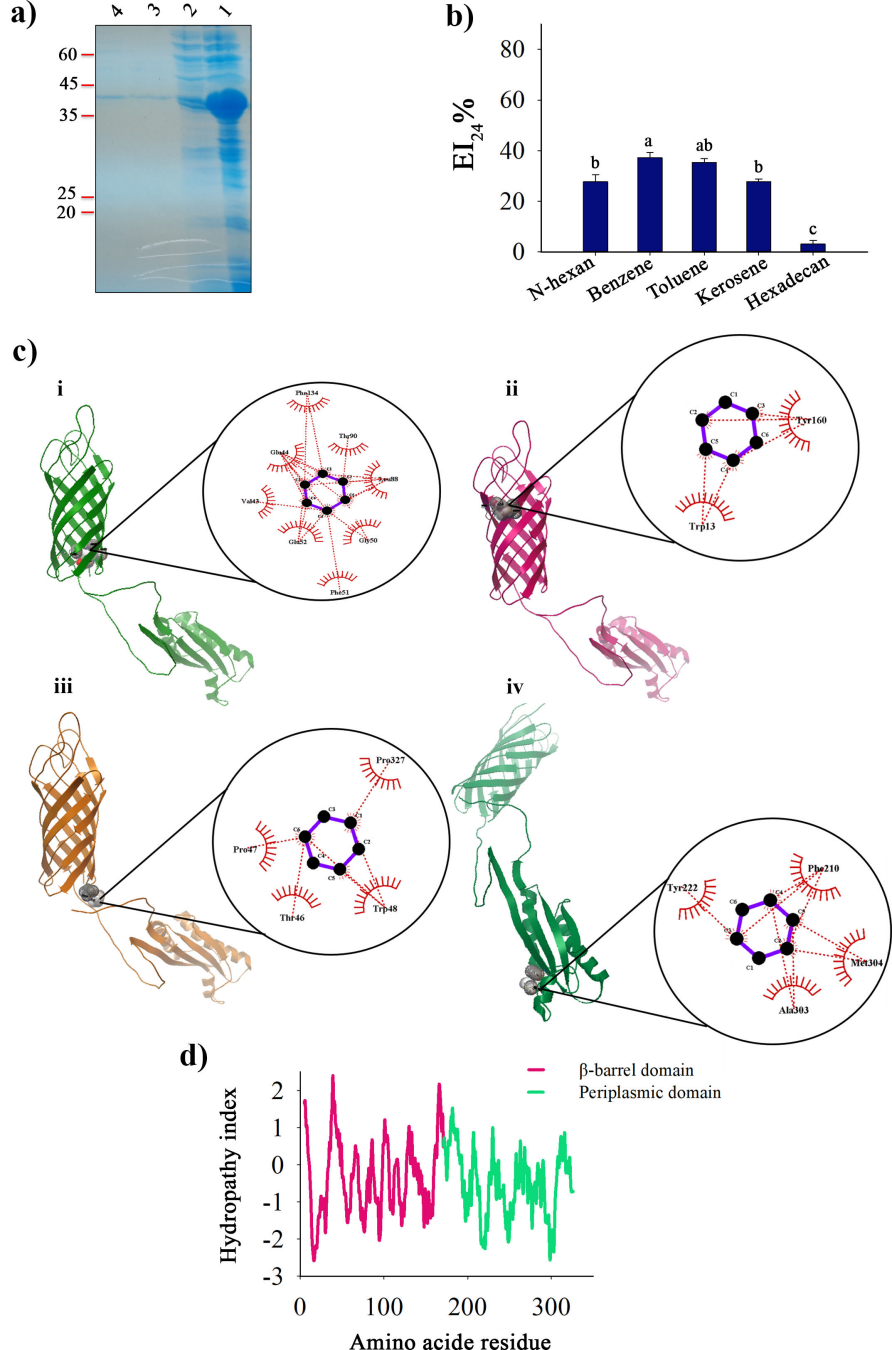
Study of full-length SA01-OmpA protein. (**a**) The SDS-PAGE analysis of full-length SA01-OmpA. Lane 1: protein fraction after 6-h induction. Lane 2: Soluble protein fraction post-sonication (crude lysate). Lanes 3 and 4: His-tag-purified SA01-ompA. (**b**) The emulsifying activity of full-length SA01-OmpA toward five hydrophobic compounds with a concentration of 43 µg mL^−1^. Different letters above the bars indicate significant differences (one-way ANOVA; Tukey test; *P* < 0.05). (**c**) Three-dimensional and two-dimensional representation of the SA01-OmpA- benzene complexes. (i) Represents SA01-OmpA and benzene complex based on grid box 1 with binding energy of −3.2 kcal/mol, (ii) Grid box 2 with binding energy of −3 kcal/mol, (iii) Grid box 3 with binding energy of −3 kcal/mol, (iv) Grid box 4 with binding energy of −3.5 kcal/mol; two-dimensional representations, in black circles, show the interaction diagram of benzene with SA01-OmpA amino acids. (**d**) Hydropathy plot analysis of full-length SA01-OmpA using the ProtScale tool. β-barrel and periplasmic domains of SA01-OmpA in the secondary structure are indicated in magenta and green, respectively.

*SA01-ompA* gene overexpression was carried out in *E. coli* strain BL21 (DE3) harboring pET26b-*SA01-OmpA*, and the protein was purified following an earlier protocol (10). Although most of the protein was insoluble in IPTG-induced cells, SDS-PAGE analysis of crude lysate revealed a narrow band corresponding to SA01-OmpA (Fig. 1a). The solubility of the recombinant protein was reduced by about 80%–90% after a year of standard storage. The bioemulsifier SA01-OmpA showed proper emulsifying activity, there are, however, significant obstacles, such as low yields and high purification costs, which prevent it from being used in real-world applications.

### Molecular docking of SA01-ompA to identify the functional part of the protein

Molecular docking can disclose structural information about binding, including the position of the ligand, protein-ligand interactions, and the residues implicated in binding (26). The AlnA protein, one of the most well-known bioemulsifiers, requires four hydrophobic areas to act as an emulsifier (27). According to earlier *in silico* investigations (10), the SA01-OmpA protein had only minor differences in those four crucial regions. The AutoDock Vina program was used to perform molecular docking based on those four hydrophobic areas. These four crucial emulsification locations were the basis of four grid boxes developed based on a study by Toren et al. (2002b), in which grid boxes 1 and 2 were found in the β-barrel domain of the SA01-OmpA protein, while grid boxes 3 and 4 were found in the periplasmic domain.

Through the refinement of the raw model retrieved from the Galaxy Refine server, five models for SA01-OmpA were generated. The best SA01-OmpA model was found to have a GDT-HA value of 0.9553, RMSD 0.386, MolProbity 2.009, a clash score of 16.3, and a poor rotamer score of 0.4. The Ramachandran plot score of the selected model was determined to be 95.7%. This model was further enhanced in quality using the YASARA energy minimization tool.

The emulsifying ability of SA01-OmpA was first examined using five hydrophobic molecules, including n-hexane, benzene, toluene, kerosene, and hexadecane to select the appropriate ligand. The best-emulsified compound at the SA01-OmpA concentration of 43 µg mL^−1^ was benzene, with an emulsion ratio of roughly 37.17% (Fig. 1b). As a result, benzene was utilized as a hydrocarbon ligand for SA01-OmpA’s molecular docking. The findings of the molecular docking and LigPlot^+^ analysis revealed that benzene, as a hydrocarbon compound, might interact with different amino acids than what was suggested by Toren et al. (2002b). Notably, the function of bioemulsifiers like SA01-OmpA depends on a good balance between hydrophobic and hydrophilic areas of the protein, not just the hydrophobic area. The ligand affinity of grid boxes 1, 2, 3, and 4 was −3.2, –3.0, −3.0, and −3.5 Kcal/mol, respectively (Fig. 1c). Also, the molecular docking results showed that the average binding energy in the grid boxes located in the periplasmic domain (grid boxes 3 and 4) is lower than in grid boxes 1 and 2 (located in the β-barrel). It indicates that the binding of the ligand to the periplasmic domain is stronger than the β-barrel domain.

The periplasmic domain’s significance in the interaction with benzene was highlighted by the molecular docking, although it does not offer extensive information about the functional part of SA01-OmpA. Because of this, *in vitro* studies are necessary to fully understand how this porin protein functions in its emulsifying activity.

### Grand average of hydropathicity (GRAVY) of SA01-OmpA

Reports claim that hydrophobic areas are necessary for bioemulsifiers to act as emulsifiers (28). The GRAVY values were obtained using the ProtParam program. The β-barrel, periplasmic domains, and SA01-OmpA protein average hydrophobicity values were −0.296,–0.545, and −0.417, respectively. The hydrophobicity of the domains in the hydropathy diagram did not significantly differ; however, the hydrophobicity of the β-barrel domain was slightly higher than that of the periplasmic domain (Fig. 1d). This cause leads to the expression of SA01-OmpA in the inclusion body, becoming a further challenge. To get past the challenges at hand, our research’s goal was to develop a protein with a better production yield and significant emulsifying activity. Therefore, with this goal in mind, two truncating variants of SA01-OmpA protein, including CT-OmpA and NT-OmpA, were developed, and a thorough analysis of the relationship between their structure and activity was conducted.

### Design of CT-OmpA and NT-OmpA truncation constructs, gene cloning, heterologous expression, and purification

**Fig 2:**
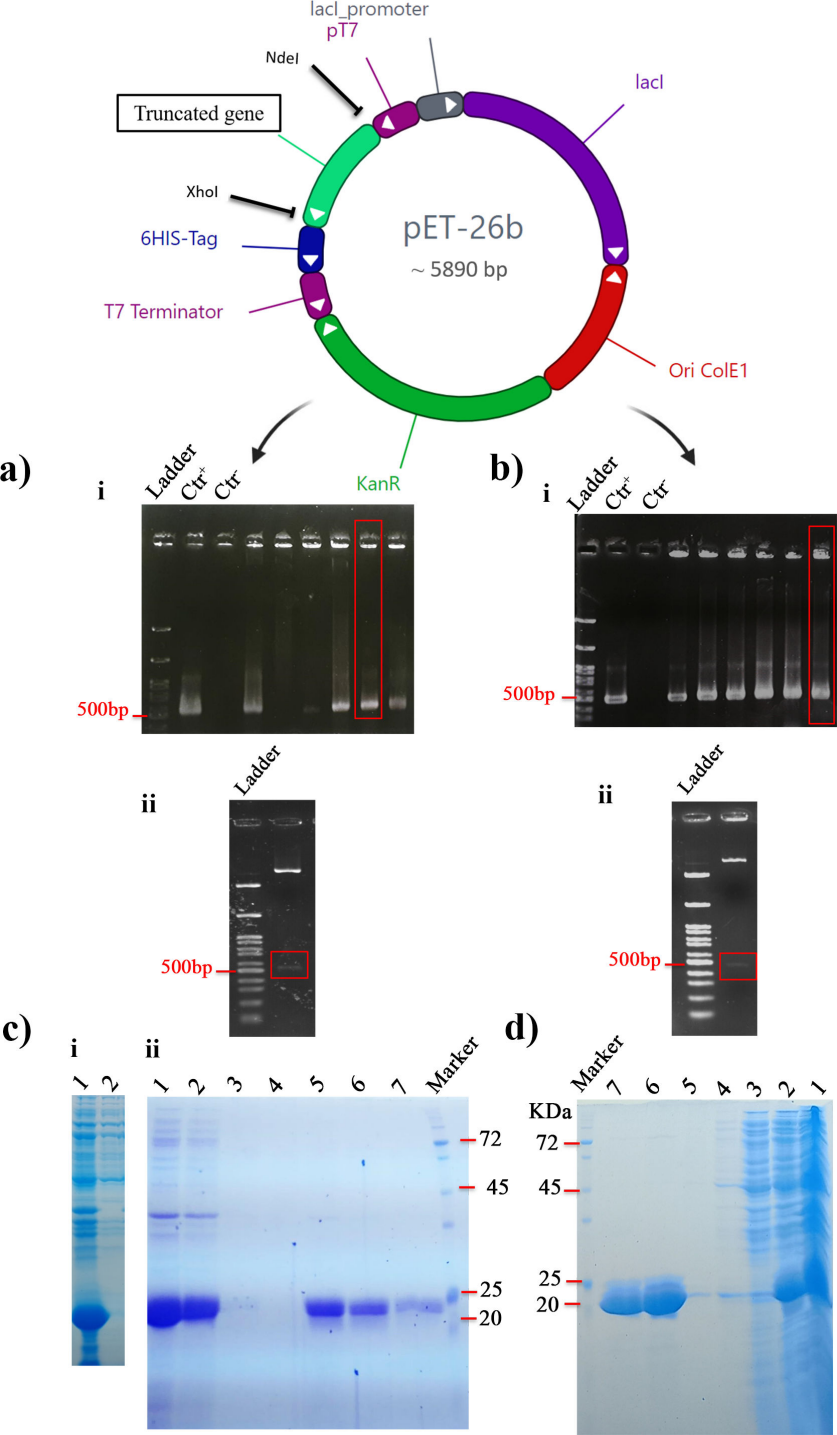
Seletion of the correct genetic construct of *CT-OmpA* and *NT-OmpA*, expression and purification of their proteins. (**a**) Demonstration of *CT-OmpA* gene cloning: (i) Colony PCR results showing a 530 bp band (in red box) confirming successful cloning. (ii) The DNA fragment of length 530 bp (red box) after double restriction-enzyme digestion confirms successful cloning. (**b**) Demonstration of *NT-OmpA* gene cloning: (i) Colony PCR results showing a 500 bp band (in red box) confirming successful cloning. (ii) The DNA fragment of length 500 bp (red box) after double restriction-enzyme digestion confirms successful cloning. Sequencing results are available in a miscellaneous file that is not for publication, (**c**) SDS-PAGE analysis of recombinant CT-OmpA, (i) Lane 1: Protein fraction after 4-hour induction; lane 2: Soluble protein fraction post-sonication (The band related to CT-OmpA protein did not appear in crude lysate), (ii) CT-OmpA solubilization of inclusion bodies (4 M urea) and purification steps, Lane 1: Supernatant after solubilization; Lane 2: Refolded soluble protein fraction; Lanes 3 and 4: Protein contents removed from the column chromatography post-dual washing with wash buffer; Lanes 5–7: His-tag-purified CT-OmpA, (**d**) SDS-PAGE analysis of recombinant NT-OmpA form, Lane 1: Protein fraction after 4-hour induction; Lane 2: Soluble protein fraction post-sonication; Lane 3: Non-His-tag proteins were removed from column chromatography; Lanes 4 and 5: Protein contents were removed after washing twice; Lanes 6 and 7: His-tag-purified NT-OmpA.

In an earlier study, a genetic construct that incorporated a signal peptide (SP) with the entire SA01-OmpA gene was designed, and it was found that SP has little impact on protein retention in the cytosol, and yet a large amount of protein continued to accumulate intracellularly as inclusion bodies. The signal peptide was thus excluded from the protein sequence (10). On the other hand, even if it was expressed in the cytosol, it was impossible to precisely analyze the protein’s emulsifying action because the peptide signal had been added to the protein sequence rather than removed. For this purpose, the genomic constructs without signal peptides were designed to get around the forgoing challenge, enabling a more accurate evaluation of the emulsifying activity of each of the variants. As shown in Fig. 2, the generation of correct recombinant genetic clones was confirmed by colony PCR, double digest, and sequencing methods (Fig. 2a and b).

Two truncating genes of SA01-OmpA, *CT-OmpA* and *NT-OmpA* were expressed in recombinant *E. coli* strain BL21 (DE3) after 4 hours of IPTG induction. Inclusion bodies were produced as a result of high expression of the CT-OmpA (C-terminally truncated SA01-OmpA). Optimizing the induction environment, such as lowering of growth temperature, reducing the shaking of the induced culture medium, inducing expression in the early-log growth phase, and using a lower concentration of IPTG, did not result in the production of the soluble CT-OmpA in *E. coli* BL21 (DE3) cells. Therefore, urea was used as a denaturing agent to solubilize inclusion bodies. The acquired data demonstrated that urea at concentrations of 4 M and higher caused CT-OmpA inclusion bodies in *E. coli* to become soluble. The recombinant CT-OmpA protein was purified following solubilization and refolding. The expected molecular mass of purified CT-OmpA was 19.2 kDa (Fig. 2c). In addition, the refolded CT-OmpA only displayed a single band in the Native-PAGE gel electrophoresis, indicating the absence of misfolded and aggregated proteins.

By contrast, the NT-OmpA (N-terminally truncated SA01-OmpA) was quickly solubilized after cell disruption *via* sonication without the need for denaturation. A Ni-NTA affinity chromatography column was used to purify the NT-OmpA. Figure 2d depicts the recombinant NT-OmpA protein as a thick band with an expected molecular mass of 18.2 kDa. The discrepancy between the expected molecular weight of proteins and their molecular weight on the gel can be attributed to the post-translational modification of protein (like glycosylation) and an abnormal migration on SDS-PAGE, which can result in unpredictable alterations to its electrophoretic mobility (29, 30).

### Investigating the activity of truncated forms of SA01-OmpA protein (CT-OmpA and NT-OmpA)

**Fig 3:**
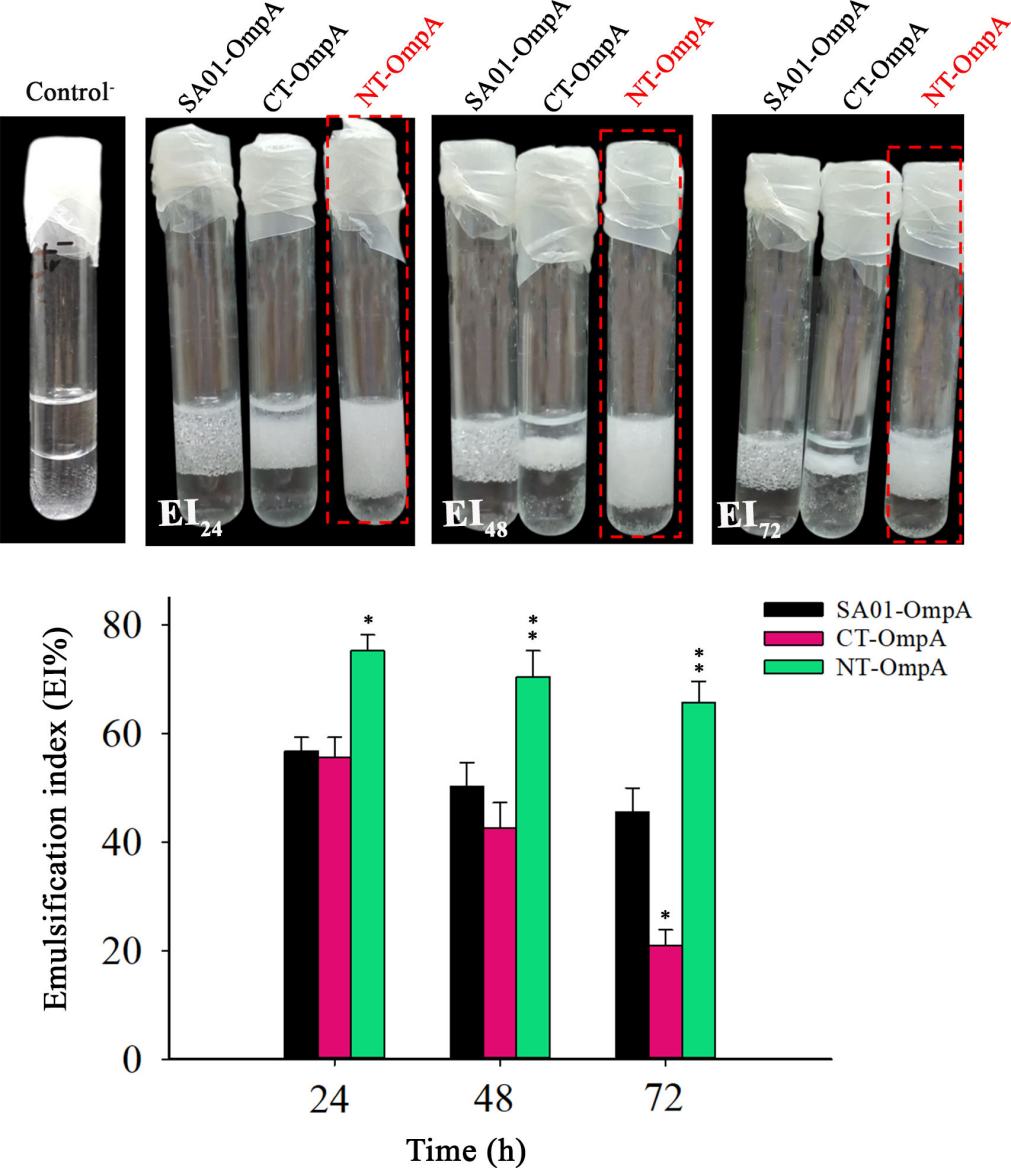
Emulsification index of SA01-OmpA and truncated proteins after 24 hours and their stability after 48 and 72 hours. All the proteins were added at a concentration of 55 µg mL^−1^ dissolved in phosphate buffer, pH 7.4. The control contained phosphate buffer without protein. An aliquot comprising 1 mL of each aqueous protein solution and 1 mL of benzene was mixed and vortexed for 2 min. Asterisks above each bar represent significant differences from full-length SA01-OmpA (one-way ANOVA; Holm-Sidak test; *P < 0.05; **P < 0.01).

The specific emulsifying activity of SA01-OmpA, CT-OmpA, and NT-OmpA (truncated proteins) was equal to 108 ± 6, 83 ± 4, and 140 ± 8 Units mg^−1^, respectively. The emulsifying activity of CT-OmpA diminished in comparison to the full-length SA01-OmpA when the periplasmic portion of the SA01-OmpA’s C-terminal end was removed, and the CT-OmpA protein was found to be in an insoluble form (inclusion bodies). Thus, the C-terminus is critical for the emulsifying activity of SA01-OmpA. Remarkably, when comparing the NT-OmpA protein to the full-length protein, deletion of the β-barrel portion from the N-terminal of SA01-OmpA increased rather than diminished the emulsifying activity of the protein. The emulsification index of proteins (EI) was measured for up to 3 days for more certainty. Figure 3 illustrates that the NT-OmpA had a greater emulsification index than the SA01-OmpA and CT-OmpA after 1 day. Interestingly, the stability of the NT-OmpA was higher than that of SA01-OmpA and CT-OmpA after 3 days, and the emulsions remained stable for several weeks (Fig. 3). In addition to the NT-OmpA’s strong emulsification activity, its solubility was also improved in comparison with the full-length protein. That is because the most truncated protein was found to be soluble in the crude lysate after sonication. As a result, it enables the high yield of pure NT-OmpA production, thereby overcoming the difficulties and expenses related to the purifying procedure. We found that the β-barrel portion is what causes SA01-OmpA’s insolubility. Because of this, the NT-OmpA’s solubility increased when the β-barrel portion was removed.

### Analysis of the structure of CT-OmpA and NT-OmpA proteins by MD simulation

**Fig 4:**
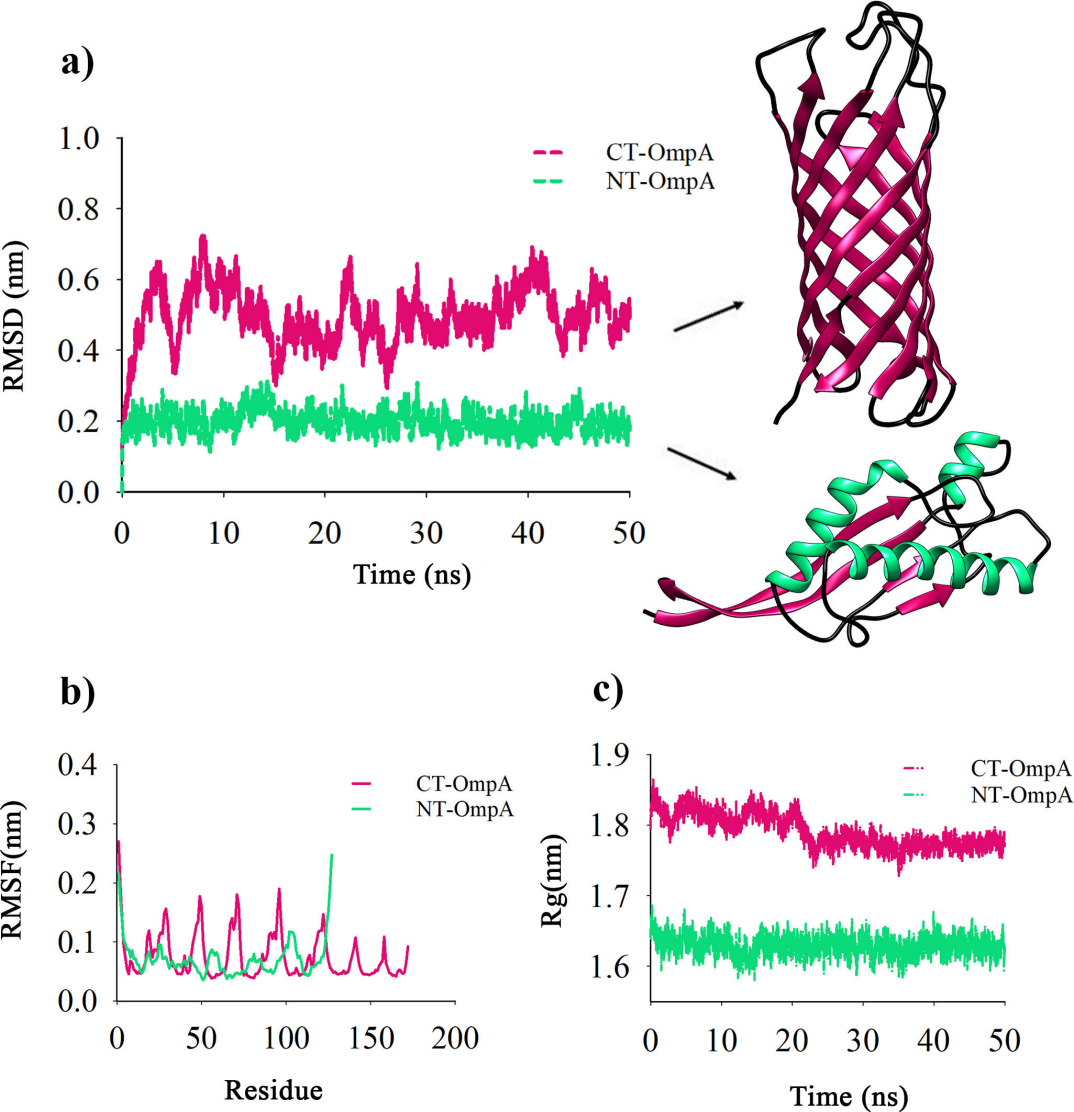
MD simulation of CT-OmpA and NT-OmpA. (**a**) RMSD plots of CT-OmpA and NT-OmpA. The RMSD plot shows that the structure of NT-OmpA was stable until the end of the simulation time. (**b**) RMSF plots of CT-OmpA (magenta) and NT-OmpA (green). (**c**) Rg plot comparison of CT-OmpA and NT-OmpA during the simulation

The truncated forms of the protein, including CT-OmpA and NT-OmpA, were subjected to MD simulation for 50 ns. In each simulation, the protein backbone’s deviation from the initial structure was plotted as a function of time to assess the structures (Fig. 4). The root means square deviation (RMSD) can be used to study the extensive changes in protein structure compared to the initial structure as well as the dynamic changes that occurred following protein modification (31). The RMSD value of the NT-OmpA was between 0.0 and 0.25 nm. The CT-OmpA showed an RSMD value in a range from 0.2 to 0.7 nm during the simulation (Fig. 4a). The findings indicated that the NT-OmpA was more stable than CT-OmpA. Using the root mean square fluctuation (RMSF) diagram, the flexibility of the backbone in the NT-OmpA was also examined compared to the CT-OmpA (Fig. 4b). In the simulation, greater flexibility is shown by a high RMSF value, whereas reduced mobility is indicated by a low RMSF value. The RMSF values of the CT-OmpA and NT-OmpA proteins were in the range of 0.1–0.2 nm, and no abnormal fluctuation was observed during the simulation. The Radius of Gyration (Rg) plot was used to assess the compactness of the truncated proteins. Although both proteins’ Rg remained steady, the shrinkage of the CT-OmpA’s Rg may be a sign of compaction and the potential for inclusion bodies formed (Fig. 4c).

### Cytotoxicity studies

**Fig 5:**
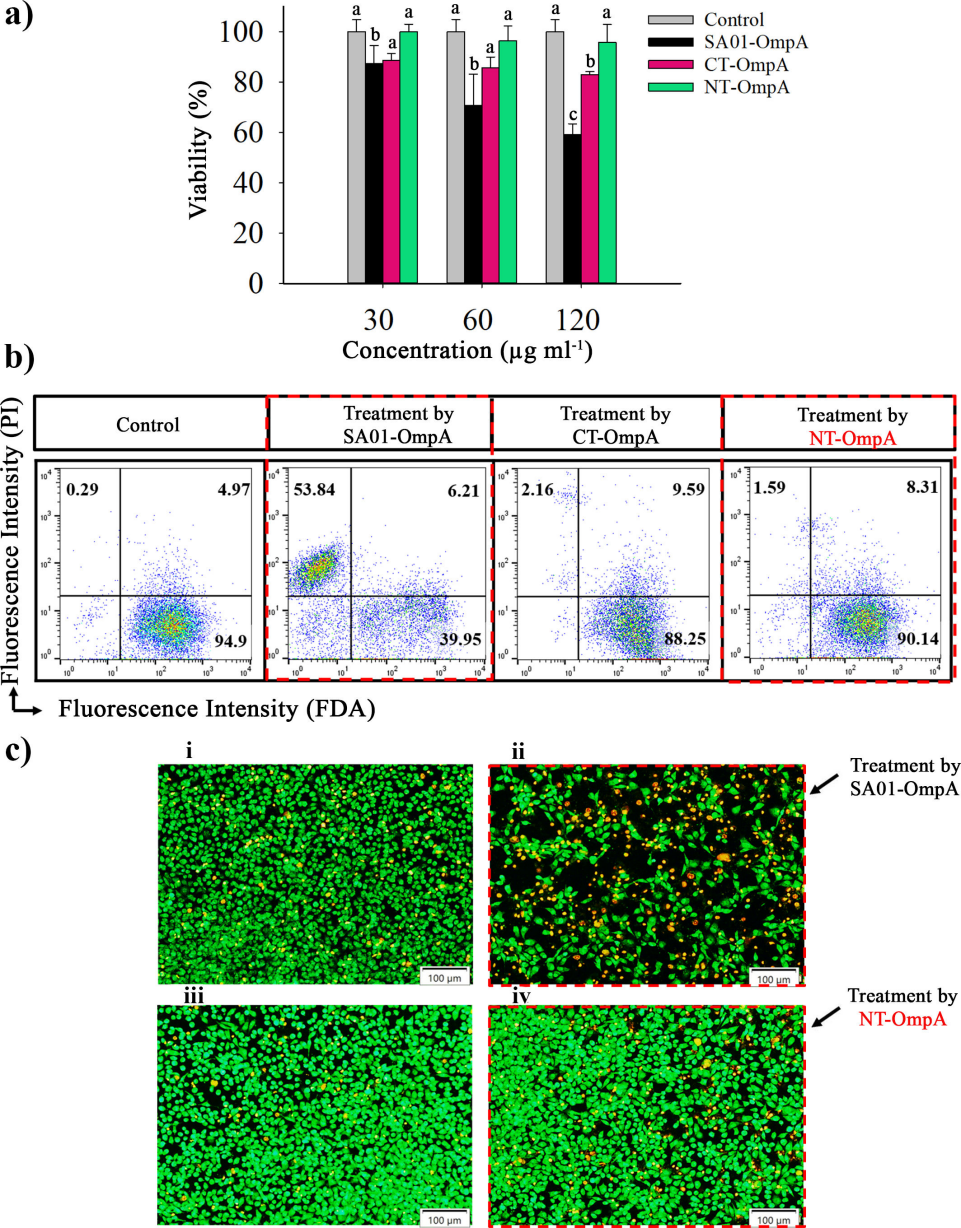
Cytotoxicity assessment of full-length SA01-OmpA and truncated proteins, (**a**) MTT assay results following L929 cell exposure to different protein concentrations. Means that do not share the same letter are noticeably different (one-way ANOVA; Tukey test; *P* < 0.05). (**b**) Flow cytometry plots show cellular response to 24-hour treatment with SA01-OmpA and truncated proteins at a concentration of 120 µg mL^−1^. The lower right quadrant shows living cells that are stained only with FDA, while the upper left quadrant depicts dead cells (stained only with PI). (**c**) AO/EB staining and fluorescence microscopy assess cell viability post-24-hour treatment with control (i), full-length SA01-OmpA (ii), CT-OmpA (iii), and NT-OmpA (iv) at 120 µg mL^−1^ concentration. Green and orange fluorescence indicate live and dead cells, respectively (scale bar = 100 µm).

The cytotoxic effects of the full-length and truncated forms of SA01-OmpA, with different concentrations, on the viability of L929 cell lines, were investigated using MTT assay after 24-hour treatments. Then the results were compared and analyzed using one-way variance; Tukey test; *P* < 0.05. Compared to the control, the cells treated with the NT-OmpA showed no discernible difference; however, the cells treated with the SA01-OmpA showed a notablly reduced viability (Fig. 5a).

Flow cytometry (FCM) was utilized to compare the populations of live and dead cells using FDA and PI dyes (32). Figure 5b shows that 53.84% of the SA01-OmpA-treated cell population was PI-absorbent and that 39.95% of the population survived, indicating the formation of an uninterrupted, unbroken membrane that was PI-impermeable. The majority of these cells died after being treated with SA01-OmpA. The majority of cells were therefore drawn to the first quadrant (Q1) of the flow cytometry plot. The NT-OmpA protein, on the other hand, 90.14% of cells survived after treatment with NT-OmpA protein. Surprisingly, PI permeability measurements from SA01-OmpA and the NT-OmpA treatments revealed a substantial difference. This finding suggests that by eliminating a portion of the original protein, the cytotoxicity of the new protein (NT-OmpA) has been decreased.

In AO/EB staining, AO-stained cells provide a uniform green fluorescence indicating living cells, while EB-stained cells provide an orange fluorescence indicating dead cells (33, 34). According to Fig. 5c, more than 90% of the stained cells in the cell population that had been exposed to the truncated proteins (CT-OmpA and NT-OmpA) exhibited a uniformly green color, similar to that of the control population. This indicates the presence of an unbroken intact membrane that was impermeable to EB. These populations contained very few orange-colored cells. Following treatment with SA01-OmpA in this case, the majority of the cell population was dead, whereas a minor percentage of cells only displayed a green hue. Low levels of green fluorescence in cell populations treated with SA01-OmpA indicate high cytotoxicity of this substance. As a result, the NT-OmpA protein was found to be completely safe and to have considerably lower cytotoxicity than SA01-OmpA.

### Cytotoxicity effects of NT-OmpA protein at different concentrations (MTT assay)

**Fig 6:**
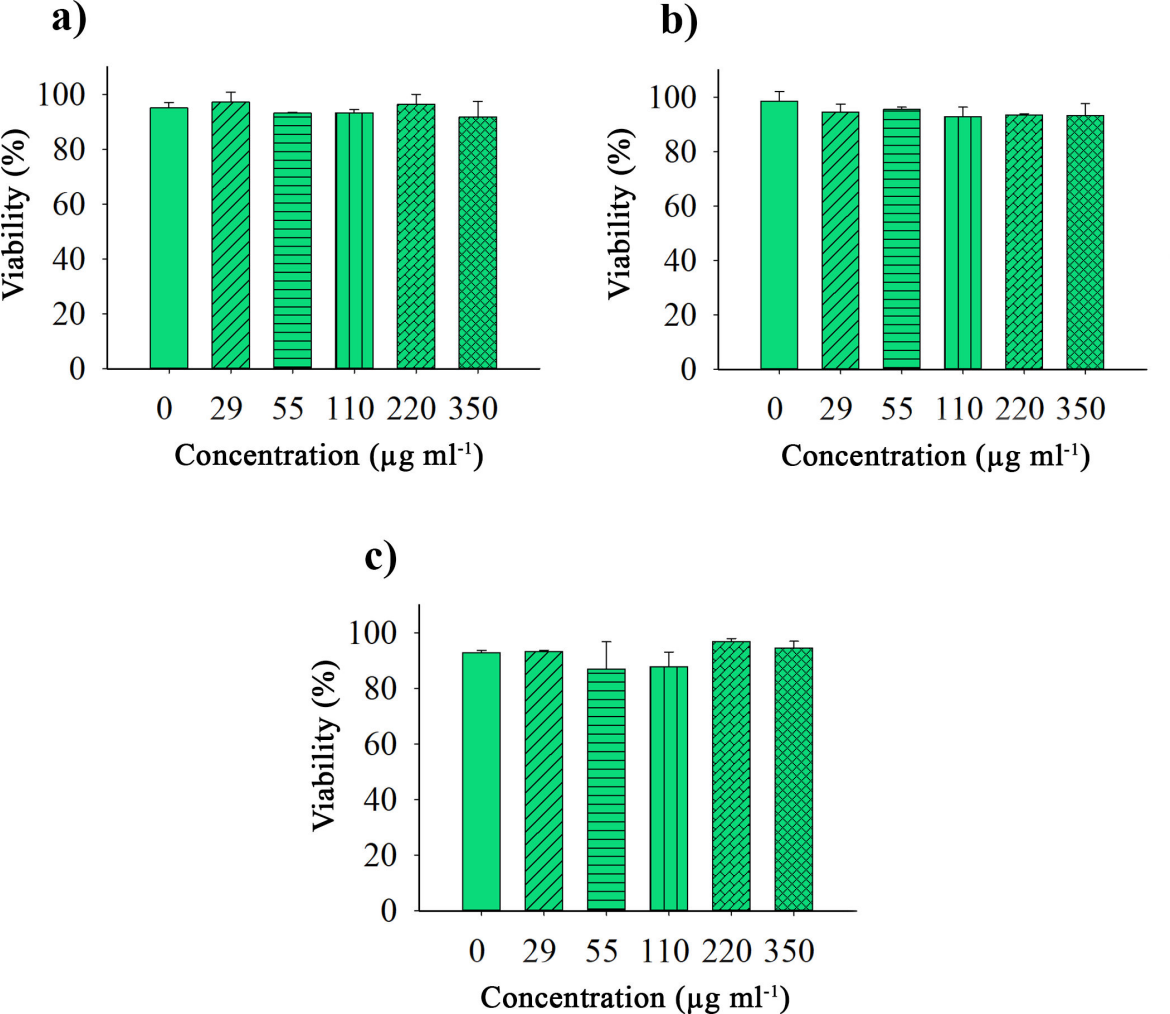
MTT assay results following exposure to different NT-OmpA protein concentrations at 24 hours (**a**) 48 hours (**b**) and 72 hours (**c**) The same control image was utilized since it was part of the same internally controlled experiment.

After it was found that truncating SA01-OmpA rendered the NT-OmpA (new protein) entirely non-toxic, the cytotoxic effects of the NT-OmpA on the viability of the L929 cell line were examined after 24, 48, and 72 hours of treatments. The results were presented as a percentage of cell viability in Fig. 6. The values obtained from the treatment of L929 cells with different NT-OmpA concentrations were analyzed using a one-way analysis of variance; Tukey test; *P* < 0.05. No significant difference was observed in all treatments compared to the control. The recombinant NT-OmpA protein appears to be safe and non-cytotoxic even at high concentrations, making it an intriguing candidate for possible application.

### Emulsification studies of NT-OmpA protein

**Fig 7:**
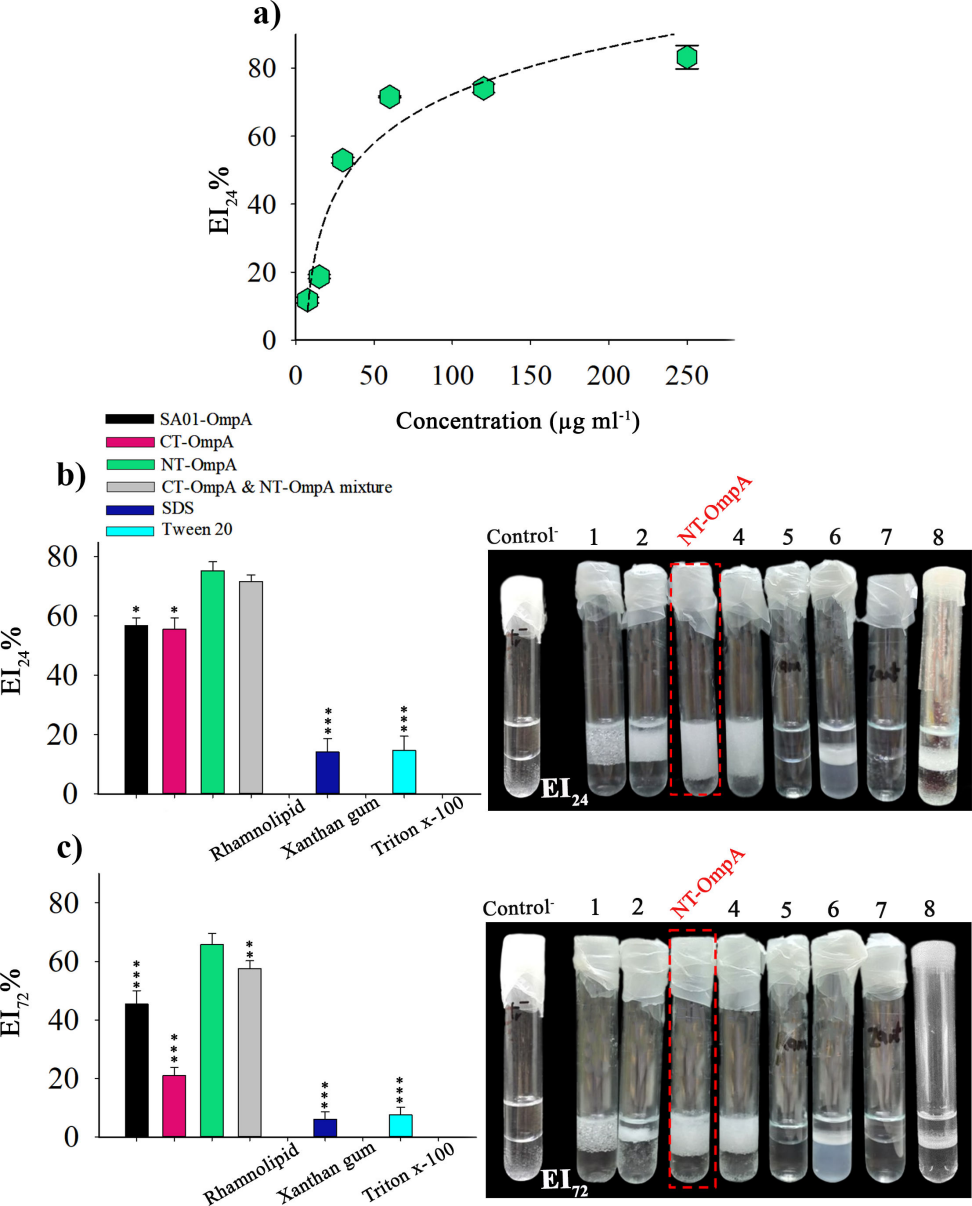
Emulsification studies of NT-OmpA protein: (**a**) Emulsifying ability of different concentrations of NT-OmpA protein using EI_24_ assay, (**b**) a comparison of the emulsification index of purified NT-OmpA with different forms of SA01-OmpA and widely used surfactants after 24 hours and (**c**) 72 hours. Some conventional surfactants, including SDS, rhamnolipid, xanthan gum, tween 20, and triton x-100 were utilized for comparison. Lane 1: full-length SA01-OmpA; Lane 2: CT-OmpA; Lane 3: NT-OmpA, Lane 4: CT-OmpA & NT-OmpA mixture; Lane 5: Rhamnolipid; Lane 6: SDS; Lane 7: Xanthan gum; Lane 8: Tween 20. The negative control sample did not exhibit any emulsifying activity after 24 hours (EI_24_) or after 72 hours (EI_72_). We, therefore, used the same image for negative control. All the proteins and surfactants were added at a concentration of 55 µg mL^−1^ dissolved in phosphate buffer, pH 7.4. Asterisks above each bar represent significant differences from NT-OmpA (one-way ANOVA; Duncan’s test; *, *P* < 0.05; **, *P* < 0.01; ***, *P* < 0.001).

The emulsification index (EI) of different concentrations of NT-OmpA protein was evaluated after 24 hours. The findings showed a relationship, although a nonlinear one, between the different concentrations of NT-OmpA protein and its ability to emulsify (Fig. 7a). To conduct further emulsification tests, the lowest protein concentration with the appropriate emulsifying ability, 55 µg mL^−1^, was chosen. Despite their harmful effects, synthetic surfactants are widely used in a variety of industries. The emulsifying capacity of the recombinant NT-OmpA protein was compared to that of SA01-OmpA protein, CT-OmpA, CT-OmpA & NT-OmpA mixture, and conventional surfactants, including rhamnolipid, SDS, xanthan gum, Tween 20, and Triton X-100. The emulsification index was measured for 1 to 3 days. According to Fig. 7b, the recombinant NT-OmpA protein had the highest emulsification index after one day equal to 75.21 ± 2.9. Xanthan gum, rhamnolipid biosurfactant, and triton x-100 did not exhibit emulsifying activity at the applied concentration. Nevertheless, NT-OmpA protein demonstrated a very strong emulsifying activity even at this low concentration (55 µg mL^−1^). Only the emulsification index (EI_24_%) of the CT-OmpA & NT-OmpA mixture resembled NT-OmpA after 1 day (Fig. 7b). Because each truncated form is more flexible on its own, it appears that the β-barrel and periplasmic domains interact with the hydrophobic compounds more favorably when they are unbound. While the emulsification index (EI_24_%) of the CT-OmpA & NT-OmpA mixture on the first day was similar to the NT-OmpA protein alone, due to the instability of the emulsions formed by the combination of CT-OmpA & NT-OmpA, emulsification index of the mixture was more different than that of the independent NT-OmpA recombinant protein within 3 days (Fig. 7c). Examining the EI_24_ % and EI_72_ % data suggests that CT-OmpA is unstable and that its presence in the CT-OmpA & NT-OmpA mixture contributed to the instability of the emulsions after 3 days (Fig. 7b and c). The collected data showed that NT-OmpA proteins have strong emulsifying properties and are highly stable.

### Emulsification of different hydrophobic compounds by recombinant NT-OmpA protein

**Fig 8:**
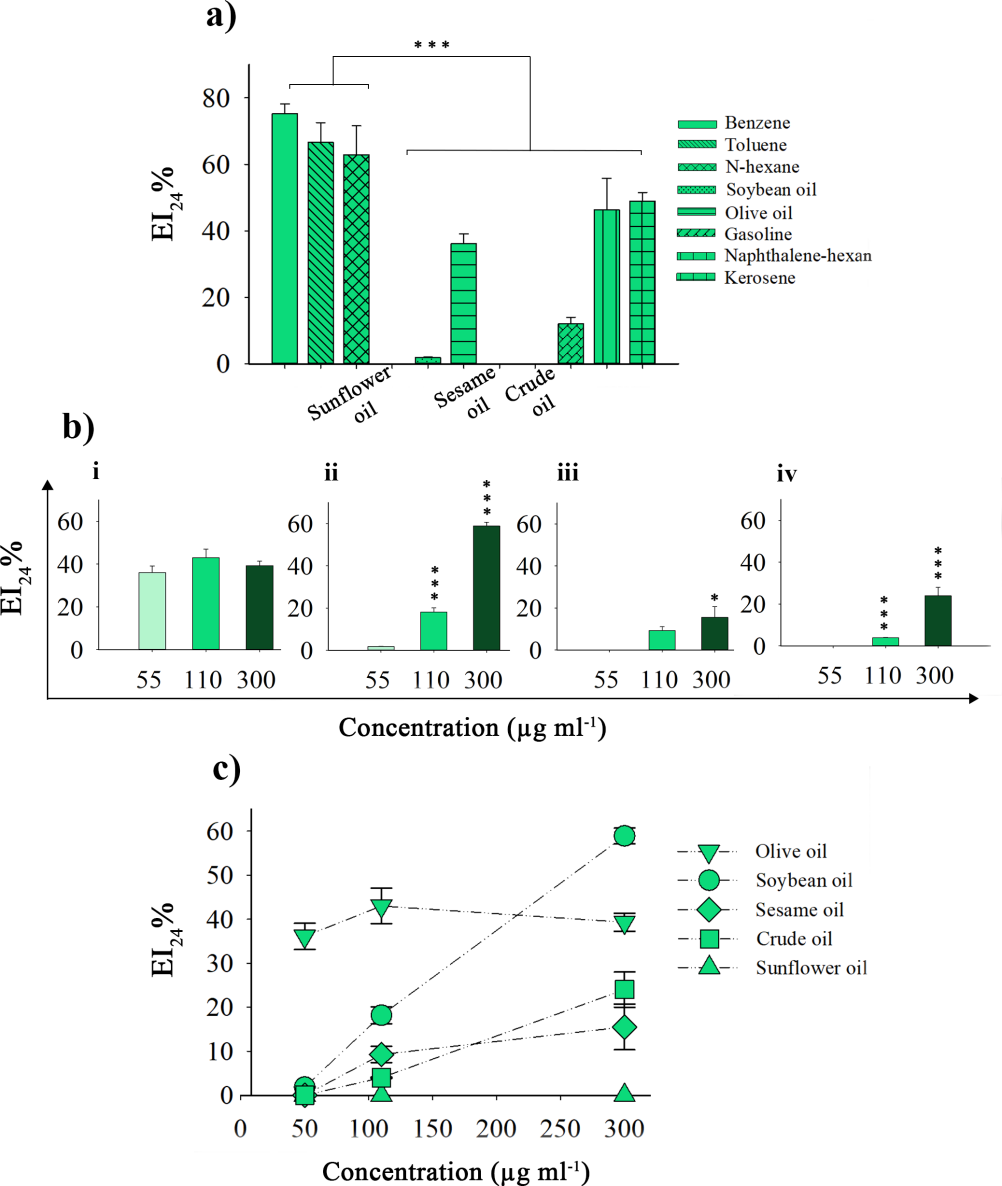
Further evaluation of the emulsifying capacity of purified recombinant NT-OmpA protein: (**a**) The emulsifying activity of NT-OmpA toward various hydrophobic compounds with a concentration of 55 µg mL^−1^, (**b**) Comparison of NT-OmpA emulsifying activity toward (i) olive oil, (ii) soybean oil, (iii) sesame oil, and (iv) crude oil at concentrations of 55 µg mL^−1^, 110 µg mL^−1^, and 300 µg mL^−1^. Asterisks above each bar represent significant differences from the lowest concentration (55 µg mL^−1^) (one-way ANOVA; Duncan’s test; *, *P* < 0.05; ***, *P* < 0.001). (**c**) Comparison of NT-OmpA emulsifying activity toward different oils in a scatter plot.

The majority of bioemulsifiers and microbial surfactants preferentially emulsify a subset of hydrophobic chemicals necessary for usage in industrial processes (15). Purified NT-OmpA protein was used to evaluate its emulsifying properties toward different hydrophobic compounds. The results showed that the NT-OmpA protein with a concentration of 55 µg mL^−1^ has a very high emulsifying ability toward hydrocarbon compounds. Benzene, toluene, and n-hexane were the best-emulsified compounds with an emulsification index (EI_24_ %) of 75.21 ±2.9, 66.6 ± 5, and 62.92 ± 8, respectively. Among the oil compounds, olive oil was also a suitable compound for emulsification, with an emulsion index of approximately 36.1 ± 3. The lowest value of the emulsification index obtained was related to soybean, sesame, and crude oils (Fig. 8a). With the increase in NT-OmpA protein concentration, the emulsification index for soybean, sesame, and crude oils increased significantly so that in a concentration of 300 µg mL^−1^, EI_24_ % for soybean oil was equal to 58.9 ± 1.8 (Fig. 8b). Therefore, with increasing concentration, NT-OmpA protein showed high emulsifying performance for a wide range of hydrophobic compounds. However, this protein showed no emulsifying effect on sunflower oil even at higher concentrations. The NT-OmpA protein demonstrated an active interaction with different hydrophobic compounds, forming stable emulsions with various hydrophobic compounds when its concentration was higher than 55 µg mL^−1^. It has previously been shown that the emulsifying activity of biopolymers is more achieved at a concentration above 300 µg mL^−1^. Hence, the high performance of NT-OmpA protein (N-terminally truncated SA01-OmpA) at low concentrations indicates its great potential for industrial applications.

## DISCUSSION

Today, emulsifiers derived from natural sources (bioemulsifiers), in recommended amounts, can be widely used in drug delivery, food processing, and cosmetic industries (35). Some bacterial outer membrane proteins deserve to be evaluated as bioemulsifiers (surface active compounds) due to their amphiphilic properties (36, 37). Although SA01-OmpA performs a wide variety of applications, its usage as a bioemulsifier is constrained due to a number of issues, including poor production and high purifying costs. Considering the limited number of proteins with appropriate emulsifying activity, the research and discovery of novel proteins with high emulsifying activity is a potential option to address these challenges (15).

For instance, in the food industry, it has been found that high-seed oil and low-seed protein in soybean are closely related with a two-nucleotide CC deletion that truncates the C-terminus of sugar transporter protein GmSWEET39, suggesting a pleiotropic influence on protein and oil content. Benefiting from the truncation technique for this protein (by elimination of 2 bp, CC) is significant in that, among the current soybean cultivars, those that have more oil and less protein are a matter of urgent necessity, addressing a critical issue that the soybean oil-extraction industries are facing (38). In addition, the comparison of the truncated and full-length forms of the *Staphylococcus aureus* protein A (SpA), a protein with pharmaceutical applications that can bind to human serum immunoglobulins and immune complexes, revealed that the truncated variant of SpA was more active in binding to IgG than full-length SpA (39). α-crystallins, as a member of the small heat shock protein family, increase the cellular tolerance of eye lenses against oxidative stress and prevent lens cataracts (40). It was found that the truncated product’s chaperone activity of αA_1-172_ was 28–46% higher than that of full-length αA-crystallin. Therefore, the presence of the truncated main product αA_1-172_ would be valuable for the αA-crystallin chaperone activities and, consequently, the eye lenses under oxidative stress (41).

Alasan, one of the best-known bioemulsifiers, is produced by *Acinetobacter radioresistens* KA53. AlnA protein is one of the components of Alasan, which is responsible for the emulsification of this complex (27). *In silico* analysis showed little difference in four key areas for AlnA emulsifying activity in SA01-OmpA protein. The interactions between fluoroquinolones, phytochemicals, and the outer membrane proteins F (OmpF) and OmpW, respectively, have previously been studied using molecular docking (26, 42). According to the key areas (grid boxes), as determined through molecular binding analysis of the SA01-OmpA protein, the periplasmic domain has the highest interaction with the ligand (Fig. 1c). Hydropathy analysis of truncated proteins by ProtScale tool showed that β-barrel domain has slightly higher hydrophobicity than periplasmic domain. Hydrophobic and hydrophilic regions in bioemulsifiers are equally essential for their emulsifying activity. This is because bioemulsifiers interact with both hydrophilic and hydrophobic regions to disperse hydrophobic substances in the aqueous medium (43). A high hydrophobicity value is not merely a measure of the higher activity of a bioemulsifier. Therefore, experimental data are needed to evaluate the emulsifying activities of proteins more accurately.

For this purpose, gene cloning, heterologous expression in *E. coli* BL21 (DE3), and purification of truncated recombinant forms of SA01-OmpA were performed using the Ni-NTA affinity chromatography column in our laboratory. Then, the production amount and emulsifying activity of truncated proteins were investigated and compared with full-length SA01-OmpA protein. It has also been reported that the recombinant PhaP protein from Alasan and Esterase from RAG-1 emulsan bioemulsifier expressed in *E. coli* leads to the formation of a highly aggregated protein referred to as inclusion bodies (44, 45). In our study, the SA01-OmpA protein (80%–90%) and the entire CT-OmpA (C-terminally truncated SA01-OmpA) were expressed as inclusion bodies in *E. coli*. On the other hand, the necessary denaturation and renaturation of inclusion body proteins increase the cost and complexity of the production process (15). By contrast, the recombinant NT-OmpA (N-terminally truncated SA01-OmpA) can be produced in a soluble form at a much lower cost. As a result, eliminating the β-barrel part from the N-terminal of SA01-OmpA could be a possible effective strategy to overcome the low production efficiency and high production costs, which hinder the practical application of SA01-OmpA bioemulsifier. Intriguingly, our findings demonstrated that deletion of the β-barrel portion from the N-terminal of SA01-OmpA did not only have discernible impact on the emulsifying activity of the NT-OmpA but also led to an increase of 18.48% in its emulsifying activity (Fig. 3). It seems that the emulsion formed by the full-length SA01-OmpA resembles that formed by large molecules, such as well-proven bioemulsifier emulsan. Furthermore, the emulsion generated by NT-OmpA is similar to low molecular weight bioemulsifiers such as terhalolipids. The recombinant NT-OmpA protein’s facile purification and significant emulsifying ability, when compared to the full-length SA01-OmpA, suggest that it has the potential to be used in various industries as a cost-effective bioemulsifier. Many studies have shown that the emulsifying activity of proteins is related to their tertiary structure (46). Hence, analyzing the structure of truncated proteins is very important to elucidate the structure-activity relationship of their truncated forms. MD studies allowed a broader range of membrane proteins (MPs) to be simulated to elucidate their structure-function relationships (47, 48). So far, the structural features of several membrane proteins (MPs) in equilibrium state, such as OmpW from *Acinetobacter baumannii* (42), PmOmpA from *Pasteurella multocida* (49), OprF from *Pseudomonas aeruginosa* (50), have been investigated using MD simulation studies. In the present study, MD simulation of truncated forms of SA01-OmpA was performed to investigate protein structural changes under equilibrium conditions. The RMSD results showed that the NT-OmpA is more stable than the CT-OmpA. While the RMSD of the CT-OmpA varied from 0.2 to 0.7 nm, the RMSD of the NT-OmpA varied between 0.0 and 0.25 nm during the simulation. Plotting the radius of gyration (Rg) of the truncated proteins individually revealed the difference in their Rg more clearly. A decrease was observed in the Rg of CT-OmpA, exhibiting the more compactness of the protein structure. Consequently, the outcomes of MD experiments supported the experimental data, emphasizing a direct correlation between CT-OmpA and its expression in inclusion bodies. The CT-OmpA that consists of eight-stranded antiparallel was fully expressed in the inclusion bodies, as confirmed by the Rg plot of CT-OmpA. Denaturation and renaturation of CT-OmpA may raise the cost of its production. This portion of the protein causes to express SA01-OmpA inclusion bodies at high levels (80%–90%) and to produce less SA01-OmpA during the purification process (60 µg mL^−1^ purified protein). Furthermore, the analysis of SA01-OmpA full-length activity and that of truncated proteins revealed that the emulsification values of CT-OmpA and SA01-OmpA were 19.66% and 18.48%, respectively, less than those of the NT-OmpA. By contrast, the recombinant NT-OmpA form, which consists of four strands and three alpha helixes, was produced in a soluble form with higher quantities (500 µg mL^−1^) and emulsifying activity. MD simulation demonstrated that the recombinant NT-OmpA is more stable than the CT-OmpA form, as indicated by experimental data. Although current research claim that high hydrophobicity is related to high emulsifying activity, our results, however, demonstrated that the recombinant NT-OmpA is a strong bioemulsifier compared to its full-length SA01-OmpA due to its suitable structural features and the proper balance between its hydrophilic and hydrophobic regions.

Understanding the potential cytotoxicity of bioemulsifiers is crucial given their growing application in important industries including food, cosmetics, and particularly pharmaceuticals (51). The highly proliferative L929 fibroblast cell line is commonly utilized in cytotoxicity testing. Fibroblast cells are the most common type of cells found in connective tissue, which, in addition to playing an active role in the synthesis and maintenance of collagen (the major structural component of extracellular matrix), modulate the behavior of adjacent cells, including migration, proliferation, and differentiation. The biological assessment utilizing fibroblast cell culture is considered a typical bioassay, providing reliable information about cytotoxicity (52). Surprisingly, the results of the FCM analysis supported those of the MTT experiment and demonstrated that SA01-OmpA, at a concentration of 120 µg mL^−1^, could make PI, a toxic chemical, more permeable (Fig. 5b). Contrarily, the L929 cells‘ decreased PI uptake following 24 hours of treatment with the recombinant NT-OmpA protein at a dosage of 120 µg mL^−1^ revealed an increase in the proportion of viable cells (>90%). Using a fluorescence microscope, the impact of SA01-OmpA and truncated proteins on the survival of the L929 cell line was visualized as anticipated, less green fluorescence was observed in cell populations treated with SA01-OmpA than in CT-OmpA and NT-OmpA proteins, indicating that eliminating a portion of the SA01-OmpA can reduce the cytotoxicity. Its enhanced cytotoxicity may be due to SA01-OmpA’s higher hydrophobicity and molecular weight in comparison to the recombinant NT-OmpA form. The hydrophobicity and amphipathic characteristics of proteins are associated with their interactions with cell membranes and cellular toxicity (17). The idea that substances with a higher molecular weight are riskier has also been put forth (18).

Efficacious emulsifying activities and the possible application of esterase AXE (as a protein bioemulsifier) from *Bacillus subtilis* CICC 20034 were previously studied. AXE showed good emulsification ability with a range of hydrophobic substances, especially benzene and short-chain aliphatic derivatives, at a concentration of 500 µg mL^−1^. Intending to develop new bioemulsifiers by combining AXE with a wide range of polysaccharides, they designed a bioemulsifier system that increased the activity of the emulsifier and further reduced the concentration of AXE required in the mixture (15). Other systems, such as RAG-1 esterase, and BD4 emulsan from *Acinetobacter calcoaceticus* like esterase AXE, cannot produce stable emulsions without combining with polysaccharides (53, 54). However, the recombinant NT-OmpA protein was able to create stable emulsions at very low concentrations without mixing with polysaccharides, and the emulsions remained stable for several weeks. To develop a bioemulsifier that was the superior of its kind, we compared the NT-OmpA protein’s emulsifying ability, at a concentration of 55 µg mL^−1^, with that of conventional surfactants. The results showed that compared to the previously studied bioemulsifiers, the recombinant NT-OmpA protein exhibits a significant emulsification activity, with an emulsification index higher than SDS, rhamnolipid, xanthan gum, Tween 20, and Triton X-100 (Fig. 7b). It was demonstrated that even at low concentrations (55 µg mL^−1^), the NT-OmpA protein exhibited an extraordinary high emulsifying activity (75.21 ± 2.9). Examining proteins’ ability to emulsify different hydrophobic substances is necessary to determine whether or not they would be appropriate for use in industrial processes (15). The NT-OmpA protein was found to be capable of creating stable emulsions using a variety of hydrophobic substances in this study. In comparison to soybean, sesame, and crude oils, it showed higher emulsifying activity with benzene, toluene, n-hexane, and olive oil. As predicted, the formation of stable emulsions rose as NT-OmpA protein content increased, especially in the case of soybean oil. Other bioemulsifiers also exhibit this effect (46). This is the first comprehensive study of its kind on protein-type bioemulsifiers. Our hypothesis that the recombinant NT-OmpA might have a better production yield and potent emulsifying activity than the full-length protein was verified by studying of the structure-activity relationship of the truncated forms of SA01-OmpA. The NT-OmpA was also completely harmless, in contrast to the SA01-OmpA protein, according to cytotoxicity assays. In addition, the recombinant NT-OmpA protein outperformed traditional surfactants in terms of emulsifying performance. The findings of our study indicate that the NT-OmpA protein (N-terminally truncated SA01-OmpA) has the potential to replace full-length SA01-OmpA as a novel bioemulsifier with significant emulsifying activity, low purification costs, and environmental compatibility. Further studies are ongoing to fully understand the functional implications of other truncated variants of the SA01-OmpA gene from cellular and molecular perspectives.

## MATERIALS AND METHODS

### SA01-OmpA production

The production of the recombinant SA01-OmpA was described in an earlier study (10). After the induction of *E. coli* BL21 (DE3) containing pET26b-*SA01-OmpA*, cells were sonicated, and SA01-OmpA was purified with Ni-NTA (Nickel Nitrilotriacetic acid) resin using standard protocols from Qiagen. Then, the purified protein was subjected to SDS-PAGE, and the concentration of SA01-OmpA was determined using a bicinchoninic acid (BCA) assay (Parstous, Iran).

### Molecular docking and grand average of hydropathy (GRAVY) of SA01-OmpA

Using the AlphaFold2 artificial intelligence (AI) program, homology modeling was carried out to forecast the three-dimensional (3D) structure of SA01-OmpA (55). The signal peptide was excluded from the protein query sequence. GalaxyRefine online server (http://galaxy.seoklab.org/) and YASARA software were used to perform structure refinement and energy minimization of 3D modeled protein structure, respectively. The crystal structure of the ligand was obtained from the PubChem database. The ligand file was changed to PDB format using Open Babel software, and the energy was minimized using Chem3D software before docking (42). Molecular docking analysis was performed using the AutoDock Vina software (56). The pdbqt file of the ligand was prepared using the AutoDock tool. Grid boxes were defined based on four hydrophobic areas that contribute to emulsification. These areas were studied by Toren et al. (27). Due to the relative similarity of SA01-OmpA protein with AlnA, these four regions were chosen as grid boxes for molecular docking of SA01-OmpA.

A protein file in the pdbqt format was made using the AutoDock Tools program for flexible body docking. The results of the docking were assessed for inter-residue interactions, binding affinity (kcal.mol^−1^), and RMSD. The binding mechanism in the benzene-SA01-OmpA complex was determined using the LigPlot + program (https://www.ebi.ac.uk/thornton-srv/software/LigPlus/). GRAVY value of SA01-OmpA and truncated proteins were calculated using the ProtParam tool (https://web.expasy.org/protparam/).

### Design of CT-OmpA and NT-OmpA genetic constructs, PCR amplification, digestion, ligation, and transformation

In a previous investigation, the whole genome of *Acinetobacter* sp. SA01 was sequenced, and a series of *in silico* analyses were carried out to precisely identify the candidate gene encoding OmpA. The recombinant *E. coli* strain DH5α-pET26b-*SA01-OmpA* was constructed in our laboratory (10). PET26b containing the SA01-OmpA gene was used as the DNA template, in the current study, for constructing truncated genes using polymerase chain reaction (PCR). For this purpose, two pairs of primers (containing NdeI and XhoI restriction sites) were designed for amplification of 530 bp *CT-OmpA* and 500 bp *NT-OmpA* genes. The *CT-OmpA* gene was isolated by PCR using a pair of primers as forward (5′-CAGTCATATGGGCGTAACTGTTACTCCATTA-3′) and reverse (5′-GTCACTCGAGAAGAACTACGTTAAGACCCG-3′), containing NdeI and XhoI restriction sites, respectively. It was amplified using Pfu DNA polymerase (Sinaclon, Iran) with denaturation at 95°C for 5 min, followed by 30 cycles of 1 min at 95°C, 30 s at 58°C, 1 min at 72°C, and finally at 72°C for 5 min. The *NT-OmpA* gene was amplified using a pair of primers as forward (5′-CAGTCATATGGGTGGTCACTTGAAACCTGC-3′) and reverse (5´-GACTCTCGAGTTGAGCTGCAGGAGCTTG-3´) containing NdeI and XhoI restriction sites, respectively, and amplified using Pfu DNA polymerase with denaturation at 95°C for 5 min, followed by 30 cycles of 1 min at 95°C, 30 s at 57°C and 1 min at 72°C, and finally at 72°C for 5 min.

The resulting amplified DNA fragments were digested with Ndel–XhoI (Fermentas, USA) and ligated to the corresponding sites of pET26b using T4 DNA ligase in 10× reaction buffer (Fermentas, USA) for 16 hours at 4°C. The consequent recombinant plasmids (pET26b-*CT-OmpA* & pET26b-*NT-OmpA*) were used to transform *E. coli* DH5α competent cells using the heat shock method. Transformants were selected on LB with the appropriate antibiotic concentration. Finally, the correct insertion of the genes into the plasmid was verified by DNA sequencing (Rajaie Cardiovascular Medical and Research Center, Iran).

### Expression of CT-OmpA and NT-OmpA proteins

Recombinant pET26bs (pET26b-*CT-OmpA* & pET26b-*NT-OmpA*) were extracted from *E. coli* DH5α and transformed into *E. coli* BL21 (DE3) cells according to the manufacturer’s recommendation. Induction for higher expression was performed using 0.5 mM isopropyl-b-D thiogalactopyranoside (IPTG) for *E. coli* BL21 (DE3) containing pET26b- *CT-OmpA* and 0.7 mM IPTG for *E.coli* BL21 (DE3) containing pET26b- *NT-OmpA*, when OD600 of bacterial culture reached in the range 0.5–0.7 (at 25°C, 180 rpm). After incubation, cells were sonicated, and His-tagged truncated proteins were purified from the cleared lysate using Ni-NTA affinity chromatography (Qiagen, Germany).

### Purification of CT-OmpA and NT-OmpA proteins

After centrifugation of crude lysate, the supernatant was loaded onto a Ni–NTA affinity chromatography column pre-equilibrated with binding buffer (50 mM NaH_2_PO_4_, 300 mM NaCl, 20 mM imidazole with pH 8). The protein was washed and eluted using imidazole washing buffer (50 mM NaH_2_PO_4_, 300 mM NaCl, 40 mM imidazole with pH 8) and elution buffer (50 mM NaH_2_PO_4_, 300 mM NaCl, 250 mM imidazole with pH 8), respectively. At the end of the procedure, the imidazole in the purified solution was removed by dialysis. The purified truncated proteins (CT-OmpA and NT-OmpA) were concentrated using a 3 kDa Ultrafiltration cartridge (Amicon Ultra-15, Merk Millipore, Germany) and preserved in phosphate buffer, pH 7.4, at 4°C. The total protein content was determined using a BCA assay. For further validation, the purified truncated proteins were subjected to SDS-PAGE (12%).

### Preventing the expression of CT-OmpA in inclusion bodies

The procedure for extracting bioactive protein from inclusion bodies is labor-intensive, and recombinant protein frequently has poor output. As the high expression CT-OmpA led to the production of inclusion bodies, a number of methods were employed to obtain soluble CT-OmpA expression in *E. coli* BL21 (DE3) cells, such as reducing the growth temperature of the induced cultures, reducing shaking of the induced cultures, inducing the protein expression in the early exponential growth phase, and inducing the culture with a lower inducer concentration. However, the results obtained indicated that the methods mentioned above did not lead to the recovery of recombinant CT-OmpA protein from the inclusion bodies. Therefore, the utilization of various denaturant concentrations, including urea, was investigated.

### Solubilization of inclusion bodies by urea

The solubilization of the inclusion body and refolding process was performed according to the previously described method (57) with minor modifications. After cell sonication, the pellet containing the inclusion bodies was washed twice with washing buffer (20 mM Tris-base, 2 mM EDTA, PH 8) and centrifuged at 9,000 × *g* for 30 min at 4°C. The pellet was resuspended in a solubilization buffer (4 M urea, 2 mM EDTA, pH 10). The suspension was incubated for 30 min and centrifuged at 9,000 × *g* for 30 min at 4°C. Then, the solution was dialyzed against a refolding buffer (20 mM Tris-base, 2 mM EDTA, and 2% vol/vol Triton X-100, pH 10) at 4°C overnight (fresh buffer was replaced thrice). Finally, the solution was dialyzed against a binding buffer to remove the detergent and loaded onto a Ni–NTA affinity chromatography column. The recombinant CT-OmpA protein was purified as described above. The sample was then centrifuged at 12,000 × *g* for 30 min (4°C) to eliminate misfolded protein aggregates.

### Emulsifying activity of SA01-OmpA and truncated proteins

The emulsifying activity was determined according to the method described by Toren et al. (58). The defined concentration of the purified CT-OmpA, NT-OmpA, and SA01-OmpA was transferred to a 10 mL glass tube containing a mixture of 20 mM Tris-HCl, pH 7.0, and 10 mM MgSO_4_ to a final volume of 1.5 mL, and then 0.02 mL of a 1:1 (vol/vol) mixture of hexadecane and 2-methylnaphthalene was added. The tubes were then vortexed at room temperature for 30 min. The turbidity was measured at 600 nm using a spectrophotometer (Bioanalytic, Jena, Germany). One unit of emulsifying activity was defined as the amount of biopolymer that yielded an A600 of 1.0 in the assay.

The EI of truncated proteins was determined using benzene and compared with full-length SA01-OmpA protein. An aliquot comprising 1 mL of each purified CT-OmpA, NT-OmpA, and SA01-OmpA aqueous solution (55 µg mL^−1^ protein preserved in phosphate buffer, pH 7.4) and 1 mL of benzene was mixed. The mixtures of benzene and aqueous solution of proteins were then vortexed for 2 min. All the samples were stored in a dark room at 25°C, and emulsification values were measured after 1 day (M & M section). The emulsification index (EI_24_ %) was calculated according to the following formula:


$$Emulsification\;index\;\left(EI_{24}\;\%\right)=\frac{\text{Height of the emulsion layer}}{\text{Height of all layers}}\times 100\%$$

The assay was performed in a identical 10 mL glass tube, and three parallel samples were measured for every emulsification value (59).

### Stability of the recombinant truncated proteins and full-length SA01-OmpA

The mixtures of benzene and each of purified CT-OmpA, NT-OmpA, and SA01-OmpA solution (55 µg mL^−1^) were vortexed for 2 min. All samples were stored in a dark room at 25°C. The stability of emulsions created by truncated proteins and SA01-OmpA was assessed *via* the EI, as described above, after 3 days.

### Comparison of emulsification index of recombinant NT-OmpA protein with other surfactants

The emulsification index of the recombinant NT-OmpA (N-terminally truncated SA01-OmpA) was investigated by emulsification index assay and compared at a concentration of 55 µg mL^−1^ with that of other commonly used surfactants, including sodium dodecyl sulfate (SDS) (Merk, Germany), xanthan gum, rhamnolipid biosurfactant (extracted from *P. aeruginosa*) (60), tween 20, and triton x-100 (Merk, Germany).

### Emulsification of NT-OmpA protein toward various hydrophobic compounds

The emulsifying ability of NT-OmpA was examined using multiple hydrophobic compounds, such as n-hexane, toluene, benzene (Merk, Germany), crude oil, soybean oil, sunflower oil, olive oil, and sesame oil by emulsification index assay. The experiment was carried out using a mixture of an aqueous solution comprising 1 mL of NT-OmpA protein (55 µg mL^−1^ protein preserved in phosphate buffer, pH 7.4) and 1 mL of the various hydrophobic compounds. The emulsification values were measured after one day.

### MD simulation of the SA01-OmpA truncated forms

MD simulation was used to evaluate the stability of truncated proteins, including CT-OmpA and NT-OmpA. Structural refinement was performed using the GROMACS software package 2018 (61). Then, the GROMOSE 54A7 force field was used to create proper topologies (62). To mimic the experimental conditions, the simulation was carried out at 343 K. The proteins were placed at the center of a dodecahedral box and solvated with the TIP3P water model while Cl− or Na+ ions were used for neutralizing. All proteins simulated under the NpT ensemble within periodic boundary conditions. The neighbor list was updated with a 10-step frequency using the grid search method. The leap-frog algorithm was used with a 2-fs time step. Protein and solvent were coupled separately to a heat bath at the desired temperature with a time constant τT = 0.1 ps applying a V-rescale thermostat. After being neutralized, the energy of the system was minimized with the steepest descent method to eliminate possible clashes and bad contacts. Subsequently, the equilibrations of proteins were done under NVT up to 100 ps at 300 K with restraint forces of 1,000 kJ mol^−1^, followed by 100 ps under NPT at the pressure of 1 bar and with restraint forces of 1,000 kJ mol^−1^ (63). The Final MD simulation was run for 50 ns with no restraint.

### Cell culture procedure

The cells used in this experiment were a continuous line of mouse fibroblast L929 cells. The cells were maintained at 37°C under 5% CO_2_ and 100% humidity in Dulbecco’s modified Eagle’s medium (DMEM) and supplemented with 10% fetal bovine serum (FBS) and 1% penicillin/streptomycin (Pen/Strep) antibiotic. L929 cells were seeded at 10,000 cells/well (96-well plate) containing 100 µl DMEM and incubated for 24 hours at 37°C under 5% CO_2_ to obtain a monolayer culture. Then, the medium was removed and 70 µL DMEM medium without FBS was added in each well, and cells were treated with 30 µL of different concentrations of proteins and incubated for 24 hours for further analysis. The experiments were carried out three times.

### Cytotoxicity assessment

#### 2,5-Diphenyltetrazolium bromide (MTT) assay

L929 cells were treated with different concentrations of proteins and incubated for 24 hours. Then, the media were removed, and the cells were washed twice with PBS buffer. Media containing 0.5 mg/mL 3-(4,5-dimethylthiazol-2-yl)−2,5-diphenyl-2H-tetrazolium bromide were added and incubated for 4 hours. After 30 min of incubation, the medium was withdrawn, and cells in 100 µL of Dimethyl sulfoxide (DMSO) were analyzed using measuring the absorbance at 570 nm in a microplate reader, and cell viability compared to control samples was evaluated (64).

### Flow cytometry analysis

After being exposed to various protein concentrations for 24 hours, cell viability was carefully assessed by flow cytometry. L929 cells were harvested by gentle trypsinization with a 0.05% Trypsin-EDTA solution, and washed with PBS buffer and suspended in a 100 µl DMEM medium. Live/dead double staining of the cells was performed with the FDA and PI. For this purpose, a working solution of FDA (10 µg mL^−1^) in PBS was prepared from stock solution (10 mg mL^−1^ in acetone), and 1 µL was added to each well and incubated for 20 min. Then, PI staining and FCM analysis were carried out, and the obtained data were examined using FlowJo Software.

### Acridine orange/ethidium bromide staining

Acridine orange/ethidium bromide (AO/EB) staining was used to detect the cytotoxic effects of the full-length protein (SA01-OmpA) and truncated proteins on the viability of L929 cells, according to the method described by Elumalai et al. (34) with minor modifications. Briefly, cells were seeded in a 24-well plate at a density of approximately 10^5^ cells/well. After 24 hours of treatment, the old medium was removed from each well, and cells were washed by PBS. Then, 250 µL of AO/EB staining solution (a mixture of dyes and PBS containing 1 µL of 5 mg mL^−1^ AO, 1 µL of 3 mg mL^−1^ EB, and 500 µl PBS) was added to each well. After 2 min, the staining solution was removed from each well and 250 µL of PBS was added to the wells. Finally, the cell viability of the stained cells was investigated using a fluorescence microscope (Nikon Eclipse-Ti, Japan).

## Data Availability

All the relevant data are contained within the article and the supplemental information.
